# Complex competition interactions between Egyptian fruit bats and black rats in the real world

**DOI:** 10.1186/s12915-025-02380-y

**Published:** 2025-10-01

**Authors:** Xing Chen, Lee Harten, Adi Rachum, Liraz Attia, Yossi Yovel

**Affiliations:** 1 School of Zoology, Faculty of Life Sciences, Tel Aviv University, 6997801 Tel Aviv, Israel; 2 Sagol School of Neuroscience, Tel Aviv University, 6997801 Tel Aviv, Israel

**Keywords:** Perceived predation risk, Non-consumptive effects, Interference competition, Behavioral plasticity, Seasonal dynamics, *Rousettus aegyptiacus*, *Rattus rattus*, Vigilance

## Abstract

**Background:**

Interspecific interactions, including competition and predation, are key drivers of ecological systems. Understanding these interactions remains challenging in the wild as it requires quantifying their effects, particularly the non-consumptive effects (NCEs) driven by predation risk. We conducted a 7-month study in a semi-natural open bat colony, monitoring interactions between Egyptian fruit bats (*Rousettus aegyptiacus*) and black rats (*Rattus rattus*) competing for food, where rats also pose a potential predation risk to the bats.

**Results:**

Video analysis revealed that bat responses to rats were fundamentally different from responses to conspecifics. The primary response was avoidance, with bat landings near food decreasing significantly when rats were present. For the 789 landings that did occur, bats showed increased vigilance and reduced foraging success, demonstrating clear NCEs. Crucially, bat foraging strategies were highly context-dependent, shifting with seasonal resource availability and rat abundance. During winter when rats were uncommon, the bats primarily employed predation risk-averse strategies (avoidance and vigilance). In spring, when rats were frequent, although there was clear temporal partitioning between the bat and the rat populations, some of the bats shifted to heterospecific interference competition, and occasionally attacked the rats to gain access to food—a behavior inconsistent with simple risk-aversion models.

**Conclusions:**

Our findings demonstrate that the bat-rat interactions are dynamically modulated by resource availability, which alters rat presence and thereby the context-dependent interplay between interference competition and NCEs. This study provides rare quantitative evidence of how behaviorally flexible animals strategically manage interference competition and predation risk based on seasonal ecological conditions.

**Supplementary Information:**

The online version contains supplementary material available at 10.1186/s12915-025-02380-y.

## Background

Interspecific interactions, particularly those involving predation [1, 2] and competition [3], are key forces driving ecological systems [1–4]. While these processes have traditionally been studied separately, growing evidence indicates that they often operate simultaneously and are modulated by the ecological context [5–7]. Seasonal fluctuations in resource availability, predator activity, or competitor density can fundamentally alter strengths of interactions and behavioral strategies [8, 9]; however, capturing these dynamics, especially their underlying behavioral mechanisms, remains challenging in natural systems.

Recent ecological theory has increasingly emphasized the importance of non-consumptive effects (NCEs), in which perceived predation risk [10] alone—without direct predation—can alter prey behavior, physiology, and morphology [2, 9–16]. These effects can cascade through communities, affecting population dynamics [11, 12] and even entire food webs [13]. However, when predation risk co-occurs with intense resource competition, prey face conflicting pressures [7]. Responses aimed at mitigating predation risk (e.g., avoidance) can conflict with the need to compete for resources (e.g., active foraging) [5, 7, 15]. It is thus critical to understand how animals navigate these conflicting demands as ecological contexts change—whether animals are dominated by NCE-related risk aversion or competition-driven resource acquisition.

Identifying the drivers of NCEs vs. competition requires understanding of the underlying mechanisms. While the behavioral responses that animals exhibit when avoiding predators (i.e., risk avoidance) may superficially resemble those exhibited during interference competition (e.g., avoiding interference competitors), they stem from fundamentally different motivations [7]. Behavioral responses aimed at mitigating predation risk (i.e., fear of death/injury) are primarily driven by the need to ensure immediate survival, often prioritizing safety over other activities. Responses to interference competition, stem from motivations such as resource defense (e.g., food-related aggressive behavior) or the establishment of dominance hierarchies, sometimes even at the cost of injury. Understanding this distinction becomes critical when attempting to identify the mechanistic origin of observed behaviors. The framework recently proposed by Wirsing et al. [9] suggests that NCEs operate through three sequential phases: risk perception, behavioral decision-making, and consequent fitness outcomes. This framework provides a structure for analyzing how animals might shift between NCE-dominated and competition-dominated strategies as ecological conditions change seasonally.

Bats globally face diverse predators [17–19], being mostly attacked in roosts [20, 21] or while emerging from them [17, 18, 21]. Predation pressure on bat populations is often significant, particularly from invasive species (comprehensively reviewed by references [17–19]). Widespread domestic and feral cats (*Felis catus* [18]) and both invasive and native rodents (e.g., *Rattus* spp.) [19, 22–24] are documented threats. The severity of such predation is highlighted by several notable cases: (1) in the Netherlands, where predation by wood mice (*Apodemus sylvaticus*) contributed to a ~ 84% of annual mortality in several species of hibernating *Myotis* bats inside specific hibernacula [22]; (2) the survival of the ground-foraging New Zealand short-tailed bat (*Chalinolobus tuberculatus*) was significantly reduced during outbreaks of introduced rats (*Rattus rattus*) and stoats (*Mustela erminea*) [23]; (3) the population of the Mexican fish-eating bat (*Myotis vivesi*) has been eradicated from several islands in the Gulf of California due to the introduction of black rats (*R. rattus*), brown rats (*R. norvegicus*), and cats (*F. catus*), especially on Mejía Island where cats had been previously eradicated, making rats the likely sole predators [24]. In the past, we also observed bat predation by rats in our colony, where black rats preyed on (i.e., killed and consumed) flightless Egyptian fruit bat pups (*Rousettus aegyptiacus*), establishing them as a local predatory threat.

Although bats comprise approximately 22% of mammalian species [25] and play diverse ecological roles, they remain relatively understudied regarding NCEs, partly due to their nocturnal and volant nature (comprehensively reviewed by reference [26]. Some evidence confirms that bats exhibit NCEs, such as adjusting roost selection in relation to surrounding cover to minimize exposure during transit [20] and altering emergence timing in direct response to the presence of diurnal raptors [21]. The Egyptian fruit bat, our target study species, also demonstrates inherent risk-averse foraging tendencies; for example, previous work [27] showed that they avoid foraging on easily accessible, low-hanging fruits, presumably to mitigate predation risk from near-ground threats, such as snakes, foxes, cats, and likely also rats [28–31].

Both Egyptian fruit bats and rats consume fruit (as a major or partial part of their diets, respectively). They naturally encounter each other on fruit trees [30, 31]. The urban environment likely increases their interaction frequency because both species feed on ornamental fruit trees and on commercial (picked) fruit. Given that rats are opportunistic predators, and that a much larger rat species (the Norway rat, is also present in our study region), it is reasonable to expect that Egyptian fruit bats (who are generally extremely hesitant [27]) would avoid contact with rats due to fear of predation. It is thus intriguing to study how these fruit bats perceive and behaviorally respond to rats—not just as competitors, but also as potential predators.

Here, we present an analysis of the complex interactions between Egyptian fruit bats and black rats. Our semi-natural, open Egyptian fruit bat colony, where bats are free to fly out to forage and return [32, 33], offers an ideal setup to examine bat-rat interactions. Daily fruit provisioning prior to sunset has created a stable foraging hotspot that attracts both resident and neighboring colony bats, as well as local rats. We (i) quantified the full spectrum of bat responses to rat presence, ranging from avoidance to vigilance during foraging (indicating NCE costs) to active interference competition (aggression); (ii) compared these responses to encounters with conspecifics to disentangle the effects driven by predation risk from those driven by competition; and (iii) assessed how bats’ behavioral strategies and overall interaction dynamics change seasonally, linking changes to resource availability.

We hypothesized that: (i) bats would perceive rats as a potential predation threat, exhibiting different responses compared to those following conspecific encounters; (ii) seasonal changes in resource availability and rat abundance would drive a shift in bat behavioral strategies, from NCE-dominated risk aversion (e.g., avoidance/vigilance) being dominant in winter to competition-driven behaviors (including aggression) becoming more prominent in spring when rat encounters are frequent; (iii) the success of bat foraging when facing predation risk would vary contextually. Through this study, we sought to quantify how complex interspecific interactions operate in a semi-natural environment, providing insights into the dynamic interplay between resource competition and predation risk.

## Results

### Bat responses to rat presence

**Fig. 1:**
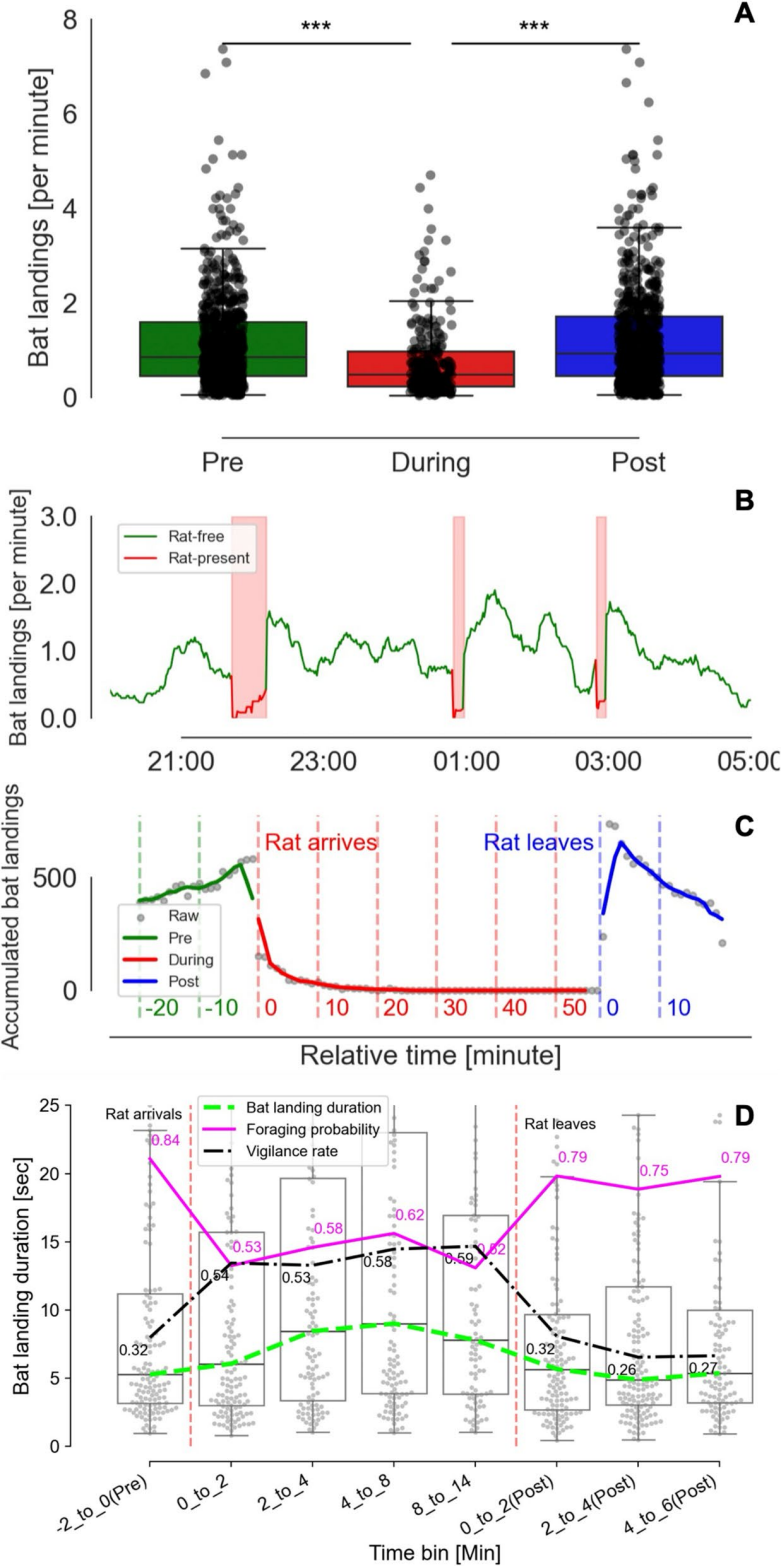
Behavioral responses of bats to rat presence. **A** Boxplots show bat landings per 1-min bins on the food platform before, during, and after rat arrivals (n_bat landings_ = 11,691, n_rat arrivals_ = 629, up to 20 min) over the 7 months from December 1, 2017, to May 31, 2018. Horizontal lines depict medians and boxes show quartiles. **B** An example of bat landings on May 20, 2018. The line shows the number of bat landings throughout the night (smoothed with a 30-min window). The green line indicates when no rat was present on the platform and the red line when a rat was present. Gray vertical bars depict the presence of one rat. **C** Seven-month accumulated bat landing events aligned according to the presence of a rat on the platform (up to 20 min before or after a rat had arrived or left). The x-axis is aligned to the arrival of the rat (with pre- and during-rat intervals depicted by green and red lines, respectively) and aligned to the leaving of the rat for the post-rat intervals (blue line). **D** Adjustments in bat foraging behavior in response to rat presence. Time course of bat foraging behaviors relative to rat arrival (red dashed line, left) and departure (right). Boxplots show landing durations for successful foraging events (green dashed line connects medians). Overlaid lines depict the foraging success rate (magenta solid line) and the percentage of landings initiated with vigilance behavior (black dash-dotted line)

Over a period of 7 months, we detected 629 events in which one or more (up to four) rats arrived on the fruit platform (with 861 rats arriving in total). During this time, we analyzed a total of 155,860 bat landings. Of these, 11,691 occurred within ± 20 min of a rat’s arrival or departure; this subset was used for most analyses (see Methods for details). Bats significantly avoided landing on the platform in the presence of rats—the rate of bats landings was significantly lower when rats were on the platform (0.26 s ± 0.59 vs. 1.11 s ± 1.14 and 1.16 s ± 1.17 landings per minute for the periods when rats were on the platform and the pre and post periods, respectively; mean ± SD, Fig. 1 A, *P*s < 0.001, Mann–Whitney *U* test). They typically flew toward the platform but, upon noticing the rat, did not land (Additional file 1: Video S1). After the rats had left the platform, the bats returned to land on it as usual with no significant difference from pre-arrival levels (Fig. 1A–C, P = 0.18, Mann–Whitney *U* test). We also documented seven events in which rats attacked bats that had landed on the platform (probably because the bats had not noticed the rat when landing; Additional file 2: Video S2).

### Complex behavioral responses and risk management in bats

Bat responses to the rats were often more complex than simple avoidance. We quantified this by analyzing 1647 landings (789 of which occurred when a rat was present), recording the landings, measuring duration of stay, assessing the bat’s success in obtaining food, and observing vigilance behaviors (Additional file 3: Video S3; see Methods).

To determine whether these behavioral adjustments stemmed primarily from interference competition or from perceived predation risk, we compared bat behavior during landings when a rat was present versus when only another conspecific bat was present (Table 1). We used conspecifics as a baseline because conspecific presence represents significantly higher interference competition compared to foraging alone (detailed in Additional file 4: Table S1). We found that when a rat was present, the bats behaved differently than when another bat was present (Fig. 1D, Table 1). They were significantly more vigilant—first scanning their surroundings more frequently before approaching the food (in 56% vs. 40%, *P* < 0.001), and for longer scanning durations (8.5 s vs. 5.2 s, *P* < 0.001). They spent more time on the platform (18.0 s vs. 11.9 s, *P* = 0.022), but obtained significantly less food (in 55% vs. 66%, *P* = 0.005). All full statistics are detailed in Table 1.

**Table 1:** Vigilance behaviors and foraging success of bats in relation to rat and bat presence with scanning/landing duration

Behavior	Metric	Rat present	Bat present	*P* value (statistic)*
**Scanning first**	Probability	56% (442/789)	40% (104/261)	< 0.001 (20.0)
	Scanning duration (seconds)	8.5 (± 17.6)	5.2 (± 13.6)	< 0.001 (1.2e6)
**Foraging success**	Probability	55% (437/789)	66% (171/261)	< 0.001 (8.0)
	Landing duration (seconds)**	18.0 (± 34.3)	11.9 (± 25.6)	0.022 (4.2e5)

*P values and their statistics (in parentheses) from χ^2^ test (with chi-squared statistic, df = 1 and *N* = 1050) for the probabilities, and Mann–Whitney *U* test (with *U* statistic) for the durations

**Only for successful foraging events

After the rat left (the “Post” bins in Fig. 1D), both landing duration and foraging success rate quickly (within < 6 min) returned to pre-rat-arrival levels (duration median of 5.2 s vs. 5.3 s, *P* = 0.250, Mann–Whitney *U* test; foraging success rate of 78% vs. 84%, Fig. 1D). This indicates the bats’ rapid reassessment of risk once the immediate threat was removed.

### Consistent caution by bats during prolonged rat presence

To assess whether the bats became more likely to land when the rats lingered on the platform, we compared the rates of bat landings in three time bins (0–3 min, 3–6 min, and 6–9 min) after rats had arrived on the platform. We found no significant difference among these intervals, with bats landing at rates of 66%, 68%, and 69% (odds ratio test, *P*s > 0.05 for 0–3 vs. 3–6 and 3–6 vs. 6–9), indicating that they remained consistently cautious whenever rats were present.

### Temporal patterns of rat activity depended on food availability

The number of observed rats on the platform was more than four-fold higher during the months April and May (54.9 events/week) compared to from December through March (12.4 events/week, *P* = 0.016 from Mann–Whitney *U* test). In addition, rats spent almost double the time on the platform per visit in April–May (5.3 min ± 0.2, mean ± SD) compared to December–March (3.0 min ± 0.2, *P* < 0.001, Table 3).

**Fig. 2:**
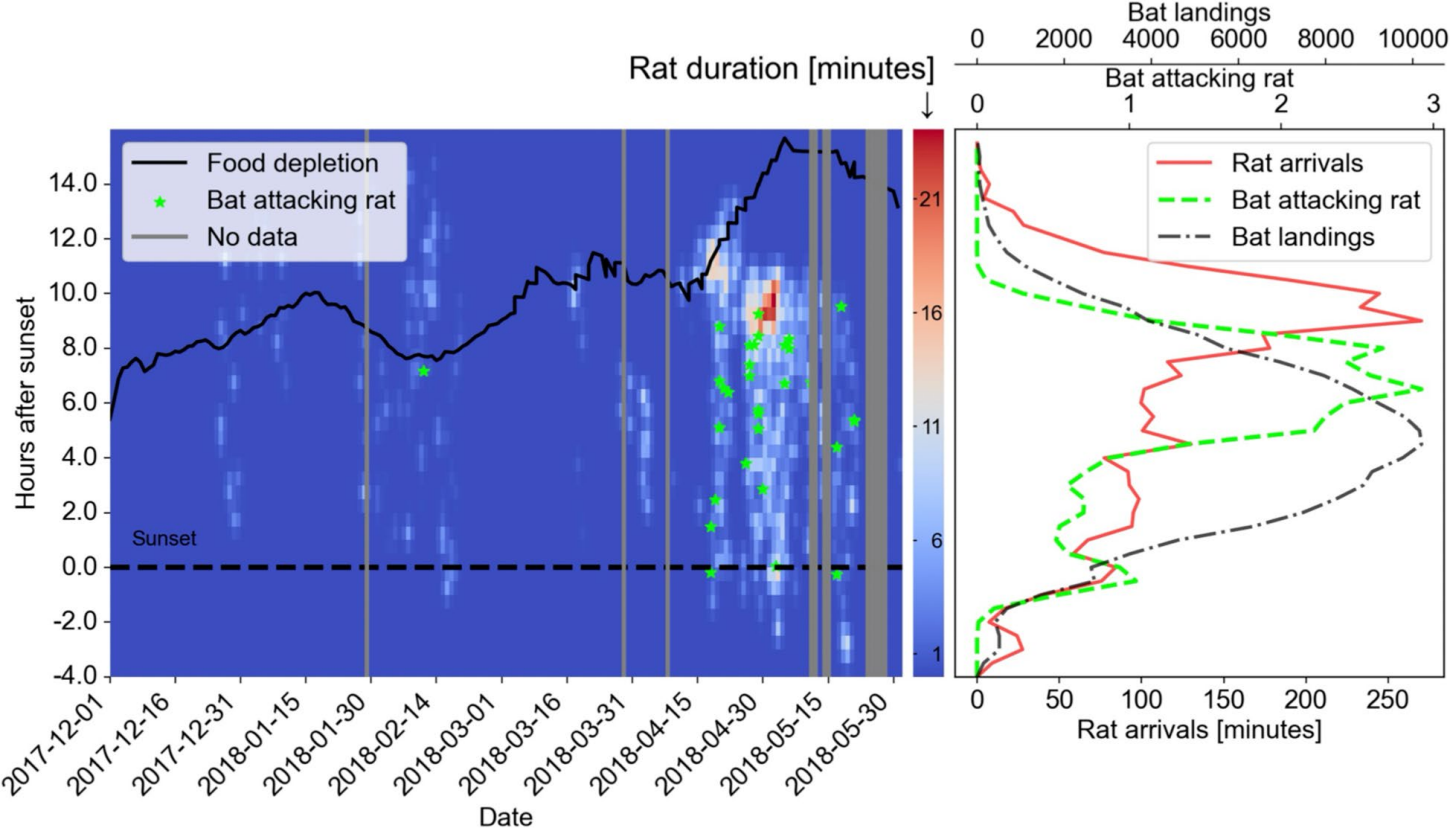
Rat-bat interactions over food. Left—A heatmap depicting rat activity on the platform over time from December 2017 to May 2018 (x-axis) and from before approximately 4 h before sunset to 16 h after sunset (i.e., approximately ~ 2:00 PM to 10:00 AM next following morning, y-axis). Green asterisks depict bat attacks on rats. The solid black line depicts the time when approximately 90% of the food had been consumed each night (primarily by bats). Gray columns depict nights with missing video. Right—Histograms of average rat activity (red solid line), bat landings (black dash-dot line), and bat attacks on rats (green dashed line), all in 30-min bins averaged over the entire period of the study

The rats’ arrival at the platform depended on seasonal food availability. As the season progressed and more external food became available, the bats did not consume all the food by the end of the night, and more rats appeared on the platform, mostly arriving toward dawn. Figure 2 presents the time of food depletion relative to sunset (black line) above a 2D distribution of rat arrival times. This figure suggests that from March onwards, food remained until morning and that, accordingly, rats mostly visited the platform in April–May, with their peak activity on the platform occurring between 9 and 9.5 h after sunset. This pattern was significant—the rats arrived significantly more often when there was more food on the platform, both late in the season and late at night (generalized linear model, GLM; Table 2).

**Table 2:** Summary of the best GLM for rat arrival number (per 30 min) on the platform

Predictor	Estimate	SE	*t*-Statistic	*P* value
Intercept	− 2.100	0.230	− 9.093	< 0.001
Food availability	0.055	0.022	2.500	0.012
Hours after sunset	0.148	0.049	3.014	0.002
Season (spring)	0.702	0.136	5.136	< 0.001
Season (spring) × hours after sunset	0.043	0.020	2.222	0.026

### Seasonal shifts in bat-rat interactions

The bats appeared to adjust their behavior according to the probability of rat occurrence. While the proportion of bat landings starting with scanning behavior did not change significantly between seasons (52% in December–March vs. 57% in April–May; *P* = 0.30, all full statistics in Table 3), the bats spent nearly three times longer scanning during these assessment periods in April–May (9.6 s) compared to December–March (3.6 s, *P* < 0.001). Moreover, in April–May, the bats successfully obtained food during 60% of landings when a rat was present, compared to only 35% success in December–March (*P* < 0.001). Furthermore, Fig. 2 shows that even in spring when rat activity increased, most bat activity occurred within 5–8 h after sunset (Fig. 2, black histogram peak in right panel) while most rat activity occurred after 9–12 h (Fig. 2, red histogram peak in right panel)—this suggests a temporal separation strategy.

**Table 3:** Seasonal comparison of bat and rat behaviors on the food platform

Category	Behavior	Dec–Apr	Apr–May	*P* value (statistic)**
Bat*	Mean bat landings per week	8.9	79.5	0.003 (17.0)
	Bat scanning first	52% (79/152)	57% (363/637)	0.300 (1.1)
	Bat scanning duration (s)***	3.6 (± 6.5)	9.6 (± 19.2)	< 0.001 (4.0e5)
	Bat foraging success rate	35% (53/152)	60% (384/637)	0 (31.1)
	Bat landing duration (s)***	5.5 (± 7.1)	15.0 (± 29.9)	< 0.001 (3.0e5)
Rat	Mean rat arrivals per week	12.4	54.9	0.026 (36.5)
	Rat residence time (m)***	3.0 (0.2)	5.3 (0.2)	< 0.001 (3.0e5)
Interaction	Bat-attack-rat events	1	30	N/A

*Only the events when a rat was on the food platform are included

**P values and its statistics (in parentheses) from χ^2^ test (with chi-squared statistic, df = 1) for the rate/probability, and Mann–Whitney *U* test (with *U* statistic) for the durations

***Only for successful foraging events

**Fig. 3:**
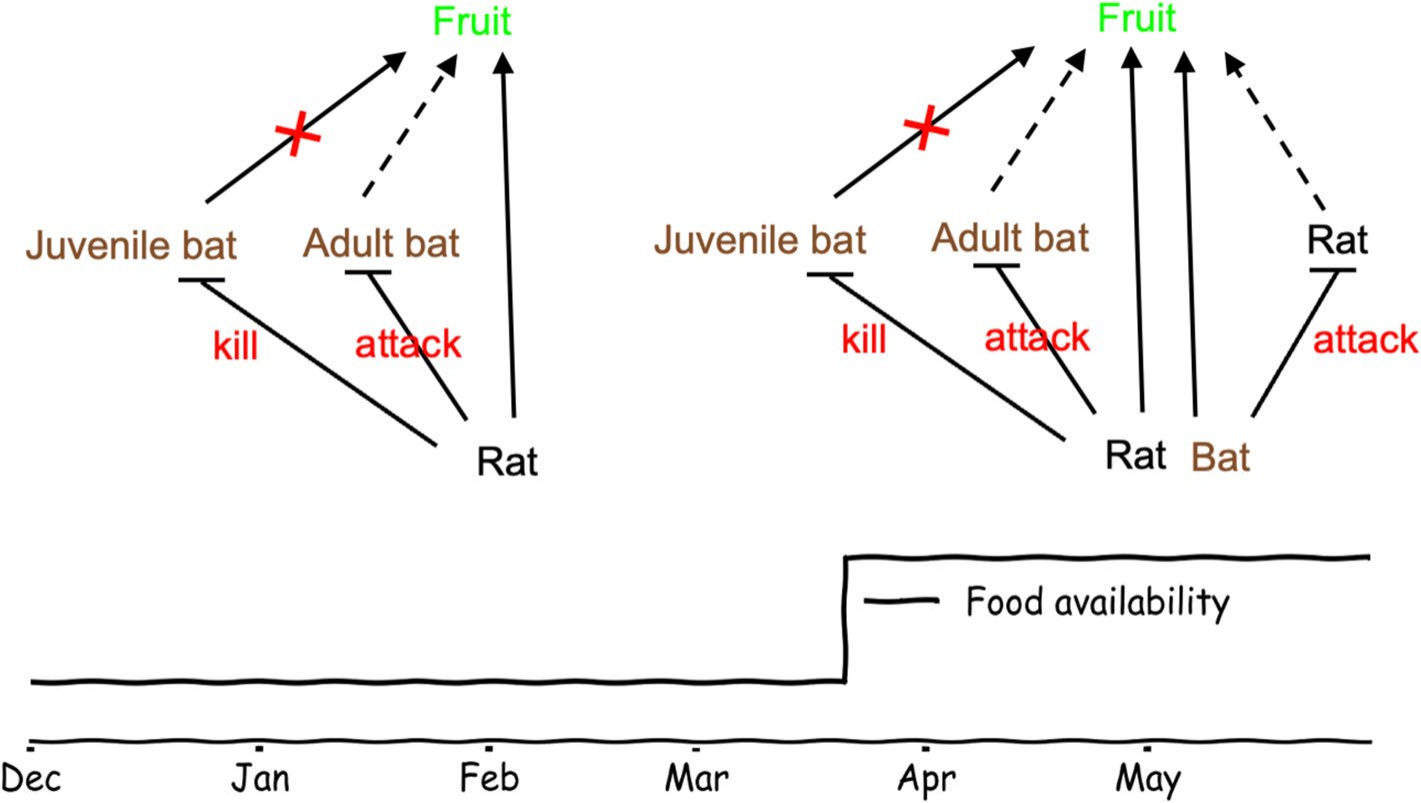
Schematic of the complex bat-rat interactions in different seasons. (Left) Winter: intense competition for fruit (solid arrows indicate consumption by bats or rats) when external resources are scarce. Bats primarily avoid the platform when rats are present. Rats are potential predators of juvenile bats. (Right) Spring: increased external food availability reduces competition. Adult bats exhibit increased interference competition, attacking rats. Solid arrows to food indicate normal consumption; dashed arrows indicate reduced consumption; red “×” symbols indicate a halt in consumption. (Bottom) Food availability increased in spring

In April–May, we also observed a new behavior in the bats: we documented 30 cases in which a bat confronted a rat and deterred it from the platform while only one such attack occurred during the months December–March (Additional file 5: Video S4; see green asterisks in Fig. 2). Attacks mostly occurred ~ 8 h after sunset (green dashed histogram in Fig. 2) when the overlap between bat and rat activity on the platform was maximal (compare red solid and black dash-dot lines in Fig. 2 histograms—right panel). All 30 bat attacks resulted in the rat immediately abandoning the food platform, while the bat either remained or flew away (Additional file 5: Video S4). Bat attacks were not more frequent when rats lingered longer on the platform (Additional file 4: Fig. S1). We summarize the complex season dependent bat-rat interactions in schematic (Fig. 3).

### Foraging success of bats when rats were present

Finally, we examined which factors affected the bats’ foraging success (i.e., obtaining food) in the presence of rats, using a logistic regression model (see Methods). The best-fit model indicated that the bats were more likely to obtain food when spending more time on the platform (Table 4). There was also a significant interaction between season and the time elapsed since a rat’s arrival, which indicates that that bats were more likely to get food when landing closer in time to the rat’s arrival time in spring as compared to in winter (Table 4). Bats landing later at night and later in the season also had a higher probability of obtaining food (the latter two predictors in Table 4). A similar significant effect of season was found when “month” was treated as a categorical variable (Additional file 4: Table S2).

**Table 4:** Summary of the best logistic regression model for the probability of acquiring food on the platform when a rat was present

Predictor	Estimate	SE	*t*-Statistic	*P* value
Intercept	− 1.283	0.244	− 5.268	< 0.001
Bat landing duration (seconds)	0.041	0.007	5.631	< 0.001
Minutes after rat arrival × season (spring)	− 0.065	0.019	− 3.401	< 0.001
Season (spring)	1.097	0.210	5.224	< 0.001
Hours after sunset	0.095	0.030	3.203	0.001

## Discussion

Our unique semi-natural environment inhabited by Egyptian fruit bats provided us with a rare quantitative glimpse into the complex interactions between two urban-dwelling generalist species. Although we did not observe rats killing adult bats at the feeding platform, we documented clear intimidation responses of the bats. The bats’ behavior significantly changed in the presence of rats in comparison to encounters with conspecific competitors. We interpret our findings through the three-phase NCE framework [9].

### Risk perception and bat landings

The first phase of the interaction requires the prey to perceive predation risk. Our findings clearly show that fruit bats perceive rats as a risk, with landing avoidance being their primary response. Bat landings decreased dramatically when rats occupied the food platform, compared to the pre- and post-rat periods during which there was only interference competition from other bats (Fig. 1 A, C; Additional file 1: Video S1 and Additional file 2: Video S2). This predominant avoidance behavior indicates successful predation risk detection—likely primarily via visual cues, perhaps supplemented by auditory information. The immediate behavioral adjustment suggests that the perceived danger often outweighs foraging requirements.

### Complexity and context dependence of the bats’ responses

The bats’ responses extended beyond simple avoidance. We observed more than 800 risky foraging events, when a bat landed with a rat on the platform.

#### Vigilance

During the 789 documented landings that occurred when rats were present, the bats exhibited significantly increased vigilance, with higher first-scanning rates and longer scanning durations compared to encounters with conspecifics (Table 1 and Fig. 1D). The increased vigilance strongly indicates that it was driven primarily by perceived predation risk, distinguishing it from baseline competitive monitoring. This vigilance likely represents a direct, measurable NCE cost—with time and energy allocated to risk assessment rather than immediate feeding. The extended scanning duration likely also reflects the increased cognitive processing, required to assess the dynamic threat posed by a rat.

#### Aggression

When examining the histograms in Fig. 2, one can see that most attacks occurred late—long after food was offered and at the peak of the overlap between bat and rat activity. Moreover, examining the videos suggests that these attacks were not consistently performed by the same individuals. Taken together, we hypothesize that as interactions become more frequent, more events result in aggression. These patterns align with Schoener’s observation [3] that interference mechanisms, such as encounter competition, often intensify with increasing competitor density. Direct aggressive encounters over resources are more likely when more individuals are trying to access those resources simultaneously.

Body size may play an important role in determining aggression responses—the two species are of similar size, and the bats can relatively easily evade rat attacks by flying away. Indeed, 30 of the 31 bat attacks were performed by adult bats, with only one by a juvenile bat; these observations highlighted the bats’ critical reliance on their flight capabilities for evasion (e.g., Additional file 2: Video S2, episode #1: after being attacked, the bat was observed to reorient at mid-fall and escape by flying away just before impact, while the rat fell to the ground). In contrast, in a few cases when a cat appeared at the entrance to the colony, the bats completely refrained from entering or exiting, suggesting fear dominance and supporting risk-calibrated decision-making (Additional file 6: Video S5 provides examples of cat predation attempts and the subsequent withdrawal of bats from entering or exiting for a short period).

### Indirect effects and ecological consequences

The above observed behaviors directly influenced ecological outcomes at the food source: bat avoidance provided rats with uncontested access to resources, and bat vigilance lowered their foraging efficiency (Table 1). Despite the clear immediate costs associated with rat presence, our observations suggest several mechanisms that may mitigate the accumulated negative impacts on bat foraging at this specific site:

1. Successful foraging under risk: Despite increased vigilance costs, the bats significantly improved their foraging success rate under rat presence from winter (35%) to spring (60%), suggesting adaptation to exploit the resources effectively even during periods of higher predation risk. A logistic regression analysis confirmed that factors such as increased time investment on the platform positively correlated with foraging success (Table 4).
2. A shift toward conflict rather than chronic suppression: In April–May, when rat activity was highest, bats sometimes initiated aggressive conflicts. This indicates that, rather than being chronically suppressed by predation risk, the bats actively engaged with and contested resources when ecological conditions favored this strategy.
3. Effective temporal management: The bats demonstrated rapid behavioral recovery, resuming normal foraging rates quickly after the rats had left (Fig. 1 C, D), indicating minimal lingering inhibitory effects on the bats’ foraging behavior.

### Seasonal context dependence and temporal food partitioning

External resource availability influenced bat-rat interactions. In spring (approximately from mid-March), fruit availability increases in the region, and bats from our colony tended to forage outdoors more frequently than in winter. Consequently, the fruit we provided was still available in the colony until dawn (Fig. 2)—when the rats were most active, resulting in temporal partitioning: bats primarily fed earlier than rats. While this temporal separation likely prevented many potential conflicts, we cannot determine if it represents an intentional avoidance strategy by either species or is simply a consequence of their differing activity schedules under these new resource conditions.

The increased food availability at the feeding station also attracted the rats for a longer duration in spring (Table 3). This elevated rat presence increased the overall bat-rat encounters and likely leading to more conflicts in spring (Fig. 2). While bats continue to exhibit NCEs related to perceived predation risk (vigilance levels remained high), their behavioral repertoire expanded due to the higher prevalence of rat encounters (Fig. 3, top right panel). Therefore, the system transitioned from being predominantly characterized by NCEs affecting bat behavior in winter, to a more complex scenario where predation, NCEs, and conspecific and heterospecific interference competition together shaped the bats’ strategies in spring.

### Broader ecological implications

Our study reflects a complex case of intraguild predation [6], a term usually used to describe complex interactions in which species that compete for the same resource also engage in predator–prey interactions. However, the case we describe is more complex than most described interactions because it includes two-way interactions. Unlike asymmetrical predator-on-predator systems dominated by super-predator effects [34], our predator–prey system was primarily driven by bat behavioral responses. When the bats’ responses were likely predominantly anti-predation avoidance (winter), NCEs dominated the system; when interference competition becomes more prominent (spring), their responses included more aggression. Our findings thus reveal a complex landscape of inter-species interactions that depended on many factors, such as encounter rates and food availability.

The bats’ strategy, dominated by avoidance and heightened vigilance at the patch, rather than completely spatial abandonment, can be seen as a form of what has been termed a “time budget shift within the dangerous patch” [35]—altering how they used the patch. Their ability to effectively utilize time as a refuge—accurately perceiving risk cessation and delaying resource exploitation until the rat left—suggests cognitive abilities beyond a simple stimulus–response mechanism. This ability enabled sophisticated risk assessment, allowing the bats to balance perceived danger against foraging needs, rather than being simply dominated by fear. This calibrated temporal awareness, coupled with strategic adjustments based on perceived risk (rat vs. cat) and competitive pressure (winter vs. spring), allows the bats to thrive in close proximity to humans and to exploit resources even in the presence of potential threats.

### Study limitations

Several limitations should be considered when interpreting our findings. While our comparison to conspecific encounters provides insight into distinguishing predation risk from competition risk, we acknowledge that, as a heterospecific species, rats may inherently elicit different competitive responses than conspecifics due to varying aggressive tendencies or persistence levels (e.g., longer residence times). While an ideal control might involve a non-predatory heterospecific exhibiting rat-like competitive intensity, identifying such a species that the Egyptian fruit bats do not fear of is challenging.

Our analysis of the 31 observed bat-initiated aggressive events is limited by the relatively low frequency of such conflict. The sample size might constrain robust statistical inference regarding the drivers of this specific competition-risk-prone behavior. Furthermore, because individual marking was not feasible for all bats in this free-ranging colony, we were unable to definitively determine whether aggression was linked to individual traits (e.g., boldness) or specific states (e.g., lactating females). Our partial examination suggests that these attacks were not consistently initiated by the same few individuals. Future research employing detailed tracking of individual behavioral histories and physiological states would enable deeper insights into risk-taking aggressive behaviors.

## Conclusions

Our findings demonstrate that interactions between Egyptian fruit bats and black rats are complex and dynamically modulated by seasonal changes in resource availability. This ecological context dictates the frequency of interspecific encounters, which in turn drives a strategic shift in the bat behavior from primarily risk-averse strategies (avoidance and vigilance) in winter to a more complex repertoire that includes interference competition (aggression) in spring. By quantifying the non-consumptive costs of vigilance and a flexible behavioral shift, our study provides rare empirical evidence for how animals navigate the conflicting pressures of predation risk and resource competition. This work underscores the importance of considering context-dependence in behavioral strategies and highlights the sophisticated mechanisms that enable urban-dwelling generalists to coexist and exploit resources in complex and dynamic environments.

## Methods

We monitored bat-rat interactions in the open colony between December 1, 2017, and May 31, 2018. Bats in the open colony were monitored with the permission of the Tel Aviv University IACUC (permit number 04–18-30).

### Video monitoring

The food bowls in the open colony were monitored using surveillance video cameras (GeoVision Inc. and Imagingsource Inc., set to a movement detection mode), enabling detailed observation of feeding behavior (as previously reported [32, 33]) and interactions between bats and rats. After manually removing the video segments triggered by humans and those segments without food on the platform, we were left with 44,469 video segments based on movement detection, totaling approximately 358 h.

### Rat detection

We manually identified 765 rat arrivals. Events were merged with subsequent events if the next one occurred within a 3-min window. This resulted in a final dataset of 629 rat events, which were used for most of analyses (Fig. 1 A and C, Fig. 2 right panel, and Table 1).

### Bat landings

We defined a bat landing event as a bat contacting the food platform and remaining on it for at least 1.5 s. This threshold was validated by manually reviewing 30 randomly selected landing events. Bat landings were detected using a machine learning model—the YOLO model [36], using the largest variant of version 11 with 56.9 million parameters—trained on approximately 25,000 manually annotated video frames. We then employed multi-object tracking [37], using the YOLO model, to track the bats. To prevent double-counting individuals, we configured the tracking model with a track buffer of 40 frames (approximately 1.6 s), enabling it to maintain the continuity of bat identities over temporal gaps. To further ensure accuracy, all detected bat landings occurring within a 20-min window before or after a period of rat presence were manually verified.

### Bat attacks

We manually identified a “bat attack” as any instance of direct physical contact initiated by a bat toward a rat at the food source. Two primary attack behaviors were observed: (1) flapping of the wings (Additional file 5: Video S4, the 11 clips, including 5–6, 8–9, 11, 13–14, 18–19, 24, and 26), which typically occurred when a bat was already at the food bowl when a rat arrived, and (2) body shoving, in which a flying, arriving bat collided with a rat already at the bowl (Additional file 5: Video S4, all other clips 1–31).

### Bat landing duration and foraging success

Bat landing duration was measured from the moment just prior to the bat landing until it left the platform. Successful foraging was defined as a landing in which the bat obtained food.

### Bat vigilance behavior

Vigilance behavior was defined as instances in which a bat, after landing on the food platform, first exhibited distinct scanning behavior, characterized by head movements directed toward the surroundings and a lack of active food handling or consumption. This was distinguished from rapid “grab-and-go” landings and from “picking” behavior, in which the bats manipulate and select food items. From this definition, we quantified two metrics for each landing: (1) the probability of scanning first, a binary measure of whether the bat’s initial action was vigilance, and (2) the scanning duration, the total time (in seconds) spent scanning during the landing event.

The bat vigilance, the bat landing duration, and foraging success were quantified for landings under three main conditions: (1) bat foraging alone (no other bat or rat visibly present at the food bowls), (2) bat landing when at least one conspecific bat was present, and (3) bat landing when at least one rat was present. Comparisons between conditions (1) and (2) were used to indicate that the conspecific interference competition was high (Additional file 4: Table S1). Thus, the conspecifics were used as a control to distinguish effects potentially driven by perceived predation risk from rats (results in Table 1). We excluded cases (*n* = 13) in which another bat and a rat were present at the same food bowl. The small number of such events prevented determination of whether the bat behavior was influenced primarily by the rat or by the competing conspecific.

### Rat-related behaviors

All rat behaviors at the feeding platform were identified and timed manually from the video recordings. A rat arrival was defined as the moment it made physical contact with the platform, and a departure as the moment it left. For analysis of rat arrival timing (Table 2), we calculated an arrival rate by summing the number of unique arrival events within 30-min bins for each night. A rat attack was defined as any instance of direct physical contact initiated by a rat toward a bat (Additional file 2: Video S2). Consecutive rat arrival events by the same species within a 3-min window were merged into a single event for analysis.

### Statistics

All statistical analyses were performed using MATLAB (version 2024b, The MathWorks Company, USA), unless otherwise specified.

We employed the Mann–Whitney *U* test to compare the number of bat landings during and before/after rat presence on the platform. To account for the different durations of these events, we compared the number of landings per minute during rat presence with landings in the pre-arrival, during, and post-departure periods using a Mann–Whitney *U* test. This non-parametric test was chosen due to the non-normal distribution of the landing data (Shapiro–Wilk test, *P* < 0.001, Python v3.11). The Mann–Whitney *U* test was also applied to compare the scanning duration (how much time bats spent scanning after landing) and landing duration (1) when a rat was present vs. when a conspecific was present, and (2) when foraging alone, and (3) the seasonal comparisons presented in Table 1, Additional file 4: Fig. S1, and Table 3, respectively.

The odds ratio analysis was applied to compare (1) the probability of a bat scanning first upon landing and the probability of foraging success between conditions (in Table 1, Additional file 4: Fig. S1, and Table 3) and (2) the probability of bats landing in successive 3-min time bins (0–3, 3–6, 6–9 min) after a rat’s arrival.

To model the factors influencing the number of rat arrivals per 30-min bin on the platform, we used a GLM with a Poisson error distribution and a log link function, fitted using the “fitglm” function in MATLAB. A logistic GLM with a binomial error distribution was used to model the probability of a bat successfully acquiring food when a rat was present on the platform. AIC-based model selection was performed by comparing models with different combinations of the fixed effects (all tested models and their corresponding AIC values can be found in Additional file 7: Table S3 and Additional file 8: Table S4).

Following model selection, we validated the assumptions for each final GLM. For the Poisson model of rat arrivals (Table 2), we checked for overdispersion by calculating the dispersion parameter (1.28), indicating that the Poisson model was appropriate. For the logistic regression of foraging success (Table 4), we assessed goodness-of-fit using the Hosmer–Lemeshow test, which indicated a good model fit of the logistic regression model (χ^2^(8) = 15.5, *P* > 0.05).

## Supplementary Information

Additional file 1: Video S1. Example of a bat avoiding landing on the platform when a rat is present.

Additional file 2: Video S2 Example of a rat-initiated attacks on bats.

Additional file 3: Video S3 Examples of bat vigilance behavior.

Additional file 4: Figure S1. The distribution of 629 rat arrival periods in relation to the distribution of 31 bat attacks. Table S1. Vigilance behaviors and foraging success of bats in relation to other bat presence and foraging alone with scanning/landing duration. Table S2 Alternative logistic regression model for bat foraging sucess, using moth as categorical predictor.

Additional file 5: Video S4 Examples of bat-initiated attacks on rats.

Additional file 6: Video S5 Examples of bat responses to a cat near the colony entrance.

Additional file 7: Table S3 Full model selection results for the GLM of rat arrival number on the platform. The best-supported model is presented in the main manuscript’s Table 2.

Additional file 8: Table S4 Full model selection results for the logistic GLM of bat foraging success when a rat was present. The best-supported model is presented in the main manuscript’s Table 4.

## Data Availability

The datasets generated and analyzed during this study are available in the Mendeley Data repository [38]: doi: 10.17632/gt7j39b2cf.2

## Abbreviations

NCEs: Non-consumptive effects
GLM: Generalized linear model
AIC: Akaike information criterion
SD: Standard deviation
IACUC: Institutional animal care and use committee
df: Degree of freedom
