# Supplementary material for: Complex competition interactions between Egyptian fruit bats and black rats in the real world
Source: BMC Biol. 2025 Oct 1;23:290. doi: 10.1186/s12915-025-02380-y (PMC12487382; doi:10.1186/s12915-025-02380-y)
Supplement: Supplementary file 4 — Additional file 4: Figure S1. The distribution of 629 rat arrival periods in relation to the distribution of 31 bat attacks. Table S1. Vigilance behaviors and foraging success of bats in relation to other bat presence and foraging alone with scanning/landing duration. Table S2 Alternative logistic regression model for bat foraging sucess, using moth as categorical predictor. [file 12915_2025_2380_MOESM4_ESM.docx]

Supplementary information


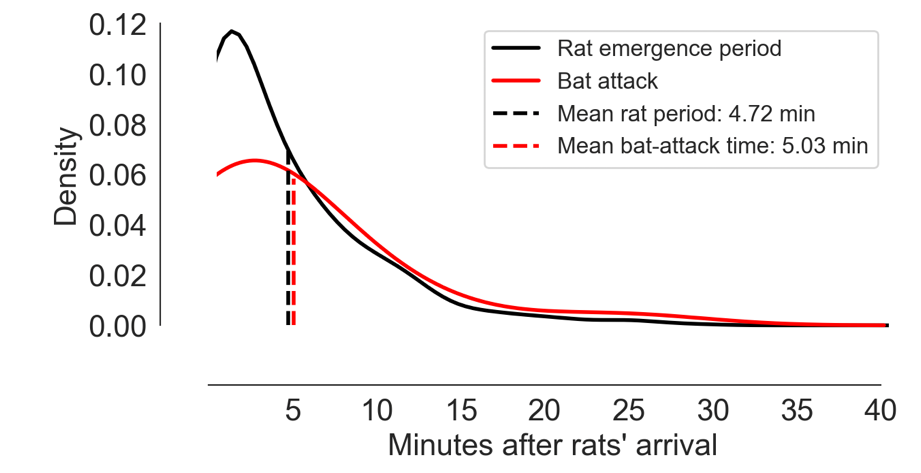


**Figure S1.** The distribution of 629 rat arrival periods in relation to the distribution of 31 bat attacks (also see video S4).

Supplementary tables

**Supplementary table 1**. Vigilance behaviors and foraging success of bats in relation to other bat presence and foraging alone with scanning/landing duration.

| **Behavior** | **Metric** | **Foraging alone** | **Bat present** | **P value (statistic)*** |
| --- | --- | --- | --- | --- |
| Scanning first | Probability | 27% (163/594) | 40% (104/261) | <0.001 (12.0) |
|  | Scanning duration (seconds) | 4.5 ($\pm$9.2) | 5.2 ($\pm$13.6) | 0.018 (7.1e4) |
| Foraging success | Probability | 85% (504/594) | 66% (171/261) | <0.001 (39.0) |
|  | Landing duration (seconds) ** | 9.9 ($\pm$ 11.7) | 11.9 ($\pm$25.6) | 0.715 (4.2e5) |

* P values and their statistics (in parentheses) from χ² test for the rate/probability, and Mann Whitney U test for the durations.

** Only for successful foraging events.

| **Supplementary table 2**. Summary of the best logistic regression model for the probability of acquiring food on the platform when a rat was present (with 'month' treated as a categorical variable) | | | | |
| --- | --- | --- | --- | --- |
| **Predictor** | **Estimate** | **SE** | **t-Statistic** | **p-Value** |
| Intercept | -0.513 | 0.430 | -1.192 | 0.233 |
| Bat landing duration (seconds) | 0.044 | 0.008 | 5.823 | <0.001 |
| Minutes after rat arrival | -0.058 | 0.018 | -3.172 | 0.002 |
| Month (Jan) | -0.362 | 0.546 | -0.664 | 0.507 |
| Month (Feb) | -1.265 | 0.471 | -2.687 | 0.007 |
| Month (Mar) | 0.126 | 0.568 | 0.223 | 0.824 |
| Month (Apr) | 0.352 | 0.411 | 0.857 | 0.391 |
| Month (May) | -0.112 | 0.425 | -0.264 | 0.792 |
| Hours after sunset | 0.092 | 0.030 | 3.105 | 0.002 |
